# Sexual behaviors and vulnerability to sexually transmitted infections among transgender women in Iran

**DOI:** 10.1186/s12905-022-01753-7

**Published:** 2022-05-14

**Authors:** Azar Nematollahi, Safoora Gharibzadeh, Maryam Damghanian, Saeid Gholamzadeh, Farnaz Farnam

**Affiliations:** 1 Department of Midwifery, School of Nursing and Midwifery, Shiraz University of Medical Sciences, Shiraz, Iran; 2 Department of Epidemiology and Biostatistics, Pasteur Institute of Iran, Tehran, Iran; 3 Nursing and Midwifery Care Research Center, School of Nursing and Midwifery, Tehran University of Medical Sciences, Tehran, Iran; 4 Legal Medicine Research Center, Legal Medicine Organization, Tehran, Iran; 5 Shahid Beheshti University of Medical Sciences, Tehran, Iran; 6 Department of Reproductive Health, School of Nursing and Midwifery, Tehran University of Medical Sciences, Tehran, Iran

**Keywords:** Sexual behavior, Sexual dysfunctions, Sexually transmitted diseases, HIV, Transgender persons

## Abstract

**Background:**

Transgender people are at serious risk for HIV infection and other sexually transmitted infections (STIs), they are four times more likely to experience HIV infection than the general population. The aim of this study was to assess sexual behaviors and vulnerability of transgender women to STIs including HIV.

**Method:**

A cross-sectional study was conducted using convenient sampling from August 2019 to March 2020 in Iran at “Support center for Iranian transgender” and “Shiraz Forensic Medicine” where transgender individuals refer to follow the steps of gender affirmation. 127 transgender women participated in this study. A researcher-made questionnaire was applied for evaluating sexual behaviors, STIs and HIV.

**Results:**

The mean age of participants and their age of sexual debut were 27.6 and 16.9, respectively. 92.1% of participants were single with experience of sex and 59.3% had one sex partner in the last 2 years. 96.9% of the participants were heterosexual with 67.2% reporting experiencing orgasm in at least 50% of their sexual intercourse. However, 42.5% reported sexual pain and the same percentage reported low or very low sexual satisfaction. About half of the participants used condoms occasionally during sex (48.7%) and the most important reason for not using condoms in most cases was not having a condom (37.9%). Some of participants had little knowledge of the symptoms (33.9%) and complications (44.1%) of STIs. Although 87.4% and 72.4% of participants had never been tested for a STI and HIV, 1.6% were HIV positive and 18.1% had a history of STIs. Also, 26% of people had undergone vaginoplasty and a significant association was observed between vaginoplasty with sexual satisfaction (*p* < 0. 01(.

**Conclusion:**

Some of transgender women in this study were involved in high-risk sexual behaviors while unaware of the signs and symptoms of STIs. Also, despite reaching orgasm in most of their sexual relationships, they had little sexual satisfaction that could probably be related to body dissatisfaction, and lack of vaginoplasty in the majority of them. The need for gender affirming surgeries and psychiatric interventions affecting body satisfaction was identified in this group.

## Background

According to the American psychological association (APA) definition, the word transgender is defined as “an umbrella term encompassing those whose gender identities or gender roles differ from those typically associated with the sex they were assigned at birth” [1]. Transgender people may face significant challenges in the type and frequency of sexual activities, pleasure, and sexual satisfaction due to the psychological effects of gender dysphoria, body dissatisfaction, and difficulty with their genitals [2, 3].

Studies conducted in 2013–2014 in Europe indicated that 46–80% of transgender people are sexually active [4, 5], most of them are heterosexual [6], prefer anal sex and have multiple sex partners [7]. On the other hand, sexually-active transgender women were less inclined to use condoms during receptive anal sex with their main partner [8] because they wanted to feel intimate with him [9].

Many transgender women are unemployed due to widespread discrimination in the workplace for transgender people [10]. The results of a study in Iran showed that nearly 87% of transgender women experienced discrimination for being transgender [11]. These people are mistreated at work, deprived of their rights, or fired [10]. Financial problems sometimes make these people involved in high-risk sexual behaviors [12] such as having multiple sex partners, unprotected sex and commercial sex workers [13]. As a result, transgender people are at serious risk for HIV infection and other STIs, and according to the Centers for Disease Control and Prevention, they are four times more likely to experience HIV infection than the general population [14]. Unsafe hormone injection, silicone use for gender affirmation and drug injections may also put them at risk for HIV infection [13, 15, 16]. As reported by the National Center for Transgender Equality in 2019, about one-quarter of transgender people in the United States failed to undergo or delayed preventative care, such as pelvic exams and STI screening test due to fear of discrimination or insulting behavior of health care providers [10].

According to official statistics reported by Legal Medicine Organizations, the prevalence of gender-affirming surgeries has significantly increased in Iran in recent decades [17]. Although any sexual relationship without marriage is legally prohibited in Iran, some studies suggest that 60% of Iranian transgender individuals have sex while they are single. The high prevalence of sexual activity in these individuals, having sex with multiple partners [18] and a high percentage of Iranian transgender women being involved in high-risk sexual behaviors, including condomless anal receptive intercourse, expose this group to serious risk of STIs including HIV, which should be given more attention [19].

In order to promote health in this vulnerable group, it is necessary to have accurate information on their sexual behavior, function and STIs. This study is part of a larger project on the reproductive and sexual health needs of transgender women. The frequency of violence, suicide and discrimination [11], as well as their quality of life, anxiety, depression and stress [20] are expressed in separate articles. In this study, sexual behavior of transgender women and their vulnerability to sexually transmitted infections were assessed. Also, the relationship between vaginoplasty and sexual variables (orgasm, sexual satisfaction, body satisfaction, and sexual pain was evaluated.

## Method

A cross-sectional study was conducted in the cities of Shiraz and Tehran in Iran from August 2019 to March 2020. It is important to note that participants were selected from a completely heterogeneous population because a large number of transgender people migrate from other cities to these two cities, especially Tehran, for better living conditions and opportunities for gender affirming care.

Participants were selected from “Support center for Iranian transgender (MAHTAA)” and “Shiraz Forensic Medicine” by convenient sampling. Among transgender people who were filed in the above-mentioned centers, only transgender women who met the inclusion criteria participated in the study. Inclusion criteria were transgender women who underwent gender affirming therapy (hormonal or surgical). Exclusion criteria were unwillingness to participate in the study and not having undergone gender affirming medical or surgical care. 185 transgender women who were filed in the above-mentioned centers were called. Forty-five women did not answer. Eight individuals failed to meet inclusion criteria and 5 women were not willing to participate in the study. Written informed consent was obtained from 127 transgender women who opted to participate in the study. All questionnaires were completed in the presence of a researcher aware of the subject of the research at the relevant center.

Unfortunately, we did not have a standard questionnaire in the local language in this study. We used a researcher-made questionnaire after evaluating its validity and reliability. Three researcher-made checklists on demographic data, sexual behaviors and STIs were used in this study. The demographic checklist included 6 items on age, marital status, education, employment, economic status, and history of vaginoplasty. Sexual behavior checklist included 10 items on sexual debut age, having sexual experience, number of sex partners, sex partner having sex with other people, sexual orientation, sexual pain, sexual satisfaction, body satisfaction, effect of body satisfaction on sexual satisfaction and prioritizing sexual behaviors in order of performing them. Transgender women were asked to prioritize sexual behaviors performed during sex from 1 to 8 (including self-stimulation, sexual fantasies, touching the genitals, romantic touching and caressing, kissing, oral sex, anal sex, vaginal sex). Behaviors with the highest frequency were ranked the first and those with lowest frequency were ranked eighth. The "not a priority" option included behaviors that had never been done before. To assess the status of STIs and HIV infection, 14 questions were designed on the knowledge on the ways of HIV transmission, recognizing the signs and symptoms of STIs, history of STIs, seeking treatment, considering oneself at risk for AIDS, the reasons for not being at risk, getting tested for STIs, rectal test, HIV test, reason for not doing HIV test, the feeling the need for condoms, the rate of condom use and the most important reason for condom nonuse. Some items were yes and no questions, some were multiple choice and some items were used to measure sexual satisfaction, body satisfaction and the effect of body satisfaction on sexual satisfaction using a 5-point Likert scale from very high to very low (in the tables, the answers are provided based on the type of question).

Validity of the questionnaires was assessed by content validity index (CVI) and content validity ratio (CVR) using opinions of 11 experts including psychiatrists, reproductive-sexual health, forensic practitioners, and psychologists. In the final questionnaire, the CVR for each question was between 0.63 to 1 and the CVI for each question was above 0.79, which was considered acceptable. Reliability was measured with a test–retest procedure on 25 transgender women with 3 weeks interval. Interclass Correlation Coefficient (ICC) of questions ranged from 0.78 to 1 and the total Cronbach's alpha coefficient was 0.8.

### Statistical methods

Continuous variables were reported as mean and Sd, question’s data were represented by number (%) for each of the questions. Cochrane-Armitage test and chi-square test were used to assess the relationship between vaginoplasty and sexual behavior variables. The between group differences in ordinal variables such as orgasm, sexual satisfaction, and body satisfaction, was evaluated by Cochrane-Armitage test for linear trend. Sexual pain in women with/out vaginoplasty was checked by chi-square test. All statistical analysis was performed in STATA (StataCorp. 2015. Stata Statistical Software: Release 14. College Station, TX: StataCorp LP.)

## Results

The mean and standard deviation of participants' age was 27.6 ± 7.3 and 48% were under 25 years old. 92.1% were single, 77.2% had high School degree and 62.2% were unemployed. 56.7% reported poor financial conditions and 74% did not have vaginoplasty (Table 1).

**Table 1:** Baseline characteristics of study participants

	Number (%)
Age groups	
< 25	61 (48
26–34	42 (33.1)
≥ 35	24 (18.9)
Education	
Primary/secondary	22 (17.3)
High school	98 (77.2)
Under/post graduate	7 (5.5)
Occupation	
Unemployed	79 (62.2)
Public sector	8 (6.3)
Private sector	40 (31.5)
Financial conditions	
Poor	72 (56.7)
Good	55 (43.3)
Marital status	
Single	117 (92.1)
Married	5 (3.9)
Divorced	4 (3.2)
In a relationship	1 (0.8)
Vaginoplasty (yes)	33 (26)

Evaluation of sexual behaviors showed that the minimum sexual debut age was 12 and the maximum was 34 with a mean and standard deviation of 16.9 ± 3.4. 92.2% had sexual experience) Sexual experience is not just about penetration, but about any behavior that makes a person feel sexual pleasure (and the majority (59.3%) had one sex partner in the last 2 years. 48.7% were unaware of their partners' potential sexual relationship with other people and 96.9% were heterosexual. 42.5% had sexual pain, 67.2% experienced orgasm in at least 50% of sexual intercourse and 41.6% had very little or low satisfaction with their sex life. About 60% of women were moderately satisfied or dissatisfied with their appearance and believed that appearance had a high or very high effect on their sexual satisfaction (Table 2).

**Table 2:** Distribution of study participants as per sexual behaviors

Variable	Number (%)
Having a history of sexual intercourse (yes)	118 (92.9)
Number of sexual partners in the last 2 years	
1 person	67 (59.3)
2–3 people	31 (27.4)
More than 3 people	15 (13.3)
Sexual partner's relationship with another person	
No	42 (37.2)
Yes	16 (14.1)
I do not know	55 (48.7)
Sexual orientation	
Heterosexual	123 (96.9)
Bisexual	4 (3.1)
Sexual pain (yes)	48 (42.5)
Experience of orgasm	
Never	9 (8)
In 25% of cases	28 (24.8)
In 50% of cases	45 (39.8)
In 75 of cases	21 (18.6)
Always	10 (8.8)
Sexual satisfaction	
Very much	3 (2.7)
Much	6 (5.3)
As medium	57 (50.4)
Low	33 (29.2)
Very little	14 (12.4)
Satisfaction with the appearance	
Completely satisfied	9 (7.5)
Somewhat satisfied	40 (33.3)
Moderate satisfaction	48 (40)
Somewhat dissatisfied	14 (11.7)
Completely dissatisfied	9 (7.5)
The effect of physical satisfaction on sexual satisfaction	
Very much	23 (20)
Much	46 (40)
As medium	40 (34.8)
Low	3 (2.6)
Very little	3 (2.6)

Evaluation of the relationship between vaginoplasty with sexual behaviors showed that only a significant association was observed between vaginoplasty with sexual satisfaction (*p* < 0. 01(, but there was no significant association between vaginoplasty with orgasm (*p* < 0. 056(, body satisfaction )*p* < 0. 36( and sexual pain )*p* < 0. 62( (Table 3).

**Table 3:** Association between vaginoplasty and orgasm, sexual pain, sexual satisfaction and body satisfaction

Vaginoplasty	Statistic	*p* value
*Sex behaviors*		
Orgasm	1.91	0.056
Sexual pain	0.49	0.62
Sexual satisfaction	− 2.53	0.01
Body satisfaction	− 0.91	0.36

The most frequent sexual behavior in transgender women was romantic touching and caressing (53.1%) and then kissing (48.7%). Oral and anal sex had the lowest frequency among sexual behaviors and had never been tried in 54% and 47% of transgender women; however, they were the third frequent sexual behavior (22.1% and 19.5% respectively). As a result, vaginal sex either did not exist or had the lowest frequency in 74.3% of all participants (This was to be expected because only 26% of transgender women had undergone vaginoplasty). 85.8% had no self-stimulation and there was no sexual fantasies or touching genitals in 70.8% of participants (Table 4).

**Table 4:** Prevalence of sexual behaviors of study participants during sexual intercourse

Sexual behaviors in order of priority	Total number = 113
Romantic touching and caressing	
First priority	60 (53.1)
Second priority	32 (28.3)
Other priorities	21 (18.6)
Kissing	
First priority	36 (31.9)
Second priority	55 (48.7)
Other priorities	22 (19.4)
Oral sex	
Has no priority	61 (54)
Third priority	25 (22.1)
The fourth priority	18 (15.9)
Other priorities	9 (8)
Anal sex	
Has no priority	53 (47)
Third priority	22 (19.5)
The fourth priority	12 (10.5)
Other priorities	26 (23)
Vaginal sex	
Has no priority	84 (74.3)
Third priority	5 (4.4)
The fourth priority	9 (8)
Other priorities	15 (13.3)
Self-stimulation	
Has no priority	97 (85.8)
Other priorities	16 (14.2)
Sexual fantasies	
Has no priority	80 (70.8)
Other priorities	33 (29.2)
Touching the genitals	
Has no priority	80 (70.8)
Other priorities	33 (29.2)

About 50% of the participants were familiar with only 1 to 4 ways of HIV transmission. 33.9% of the participants did not know any of the symptoms and 44.1% of them did not know any of the complications of STIs. 88.2% of participants did not consider themselves at risk of HIV infection, and their most important reasons for not being at risk was that they were not having sex (36.5%) or had only one sex partner (34.8%). 87.4% of participants have never undergone STIs screening, about 90% have never had a rectal exam for genital warts, and 72.4% have never had an HIV test. The majority of participants cited they failed to undergo tests because they think that are not at risk (76.1%). About 18% of participants had a history of STIs and 82.6% of them had been treated for their STIs. In the sample under study, 2 participants were HIV positive (1.6%) and 2 participants had genital warts (1.6%).

The majority of participants confirmed the need to use condoms during all types of sex (about 45%) and about one third of participants mentioned it necessary only for vaginal sex (33%). About half of the participants used condoms occasionally during sex (48.7%) and the most important reason for not using condoms in most cases was not having a condom (37.9%) or their sexual partner not accepting to use one (33.7%) (Table 5).

**Table 5:** Distribution of study participants as per sexual transmitted infections (STIs) and HIV

Variable	Number (%)
Awareness of transmition ways of HIV	
1 or 2 ways	28 (22)
3 or 4 ways	38 (30)
5 or 6 ways	24 (18.9)
All the ways	37 (29.1)
Awareness of symptoms of STIs	
Not at all	43 (33.9)
1 or 2 symptoms	51 (40.2)
3–4 symptoms	24 (18.9)
All the symptoms	9 (7)
Awareness of complications of STIs	
Not at all	56 (44.1)
1 or 2 complications	61 (48)
3–4 complications	10 (7.9)
Do you consider yourself at risk for AIDS?	
Yes	13 (10.2)
No	112 (88.2)
I do not know	2 (1.6)
Reason for not being at risk of AIDS	
No sexual contact	42 (36.5)
Regular use of condoms	8 (6.9)
Having only one sexual partner	40 (34.8)
Do not use a common needle	25 (21.8)
Test for STIs (No)	111 (87.4)
Rectal exam for HPV (No)	114 (89.8)
Test for HIV (No)	92 (72.4)
Reason for avoiding HIV test	
I do not consider myself in danger	83 (76.1)
I'm afraid and I do not want to know	11 (10.1)
Fear of losing personality	9 (8.3)
All 3 items	6 (5.5)
Do you have a history of a STI? (yes)	23 (18.1)
Have you been looking for a cure for STI? (yes)	19 (82.6)
Need to use a condom	
Anal sex	7 (5.5)
Vaginal sex	42 (33)
Oral sex	2 (1.6)
Anal and vaginal sex	17 (13.4)
All sex	57 (44.9)
I do not know	2 (1.6)
Frequency of condom usage	
Always	20 (17.7)
Sometimes	55 (48.7)
Never	38 (33.6)
Reason for not using condom	
Not having a condom	36 (37.9)
Decreased sexual pleasure	22 (23.1)
Sexual partner rejection	32 (33.7)
Alcohol and drug abuse	2 (2.1)
It was not necessary	3 (3.2)

## Discussion

This study was conducted to evaluate the sexual behaviors of transgender women and their vulnerability to STIs including HIV.

Results of the study on sexual behaviors of 127 transgender women showed that although 92% of them were single at the time of the study, about 93% had sex experience in their life. Although the majority of participants had sex with a single partner for the past 2 years, it is important to pay attention to the rest 40.7% who had more than 2 partners in the last 2 years. In Iran, sex is legally allowed only in marriage, but transgender people can rarely get married. Having extramarital sex, exposes these people to high-risk behaviors such as multiple sexual partners and STIs including HIV. This conclusion seems plausible by looking at demographic characteristics such as 62.2% unemployed and inappropriate financial situation in 56.7% of participants. In a study by Clements-Nolle, 80% of transgender women had sex in the last 6 months and 37% had sex with more than 10 people [21]. In the study by Herbst, 31.7% of transgender women had multiple sex partners [22].

In the present study, sexual interests of transgender women were similar to those of Cisgender women in many ways [23, 24]. Romantic touching and caressing (53.1%) and then kissing (48.7%) were the first and second sexual behaviors and preferences in transgender women. Naturally, due to the absence of vagina in 74% of the participants in this study, vaginal sex, despite their strong desire, was less prevalent in their sexual relations and oral and anal sex were inevitably the third sexual behavior with a frequency of 22.1% and 19.5%. The frequency of oral and anal sex in Iranian transgender women seems lower relative to other transgender women, which is consistent with the experience of these behaviors among most Iranian cisgender women [23]. By contrast, in the study by Sinha in India, all transgender participants had receptive anal sex and 73.3% had receptive oral sex [25]. In his study, 70% of participants had sex for money and therefore, they were forced to have any kind of sex, including anal sex. Other patterns of sexual behavior, such as very low preference for fantasy or masturbation, were consistent with patterns of sexual behavior among Iranian cisgender women [24].

Although transgender women had similar interests to Cisgender women, there were significant differences between them in achieving orgasm. In this study, about 67.2% of transgender women reached orgasm in at least 50% of sexual intercourse and only 8% of them did not experience orgasm, which is quite significant compared to the prevalence of orgasmic disorders (37%) [26] and anorgasmia (26%) in Iranian cisgender women [27]. Also the results of this study showed that vasinoplasty had no effect on achieving orgasm in transgender women.

It should be noted that despite reaching orgasm, transgender women did not report high sexual satisfaction and 41.6% of them reported low and very low satisfaction with their sex life. This finding is important from several perspectives. First, consistent with other studies, this finding shows that reaching orgasm does not necessarily imply high sexual satisfaction, and sexual satisfaction in women is largely influenced by other factors such as satisfaction with sexual partners [28, 29] and body satisfaction [30, 31]. In this study, vasinoplasty showed a significant association with sexual satisfaction. On the other hand, about 60% of people were moderately satisfied or dissatisfied with their appearance and believed that appearance has a great impact on their sexual satisfaction. As the results of studies have shown, although medical interventions (hormone therapy and surgery) have a positive effect on sexual feelings of transgender people, but body satisfaction, especially in transgender women, plays a more important role. Hormone therapy is more about satisfaction with the whole body, while genital surgery further contributes to genital satisfaction [2, 32]. Therefore, efforts should be made to nurture a positive body image in them.

Another reason for low sexual satisfaction in transgender women is the significant prevalence of sexual pain in them. The frequency of sexual pain in transgender women was 42.5% which is significant compared to the prevalence of severe (10.5%) and moderate dyspareunia (25.8%) in Iranian cisgender women [33]. At the same time, due to the absence of vaginoplasty in 74% of participants, they had to have anal sex which was described as painful and unpleasant. On the other hand, in this study, no significant association was observed between vaginoplasty and sexual pain.This shows that in order to improve the sexual health of transgender women, more useful and less complicated surgical methods are required, and special attention should be paid to sexual counseling, especially with their sex partner.

In addition, low sexual satisfaction in transgender women despite reaching orgasm can be attributed to common sexual scripts in the society that generally consider sex to be penetration-based, and if absent, sexual satisfaction is overshadowed by dyspareunia or absence of vagina. With proper culture development and sexual counseling, however, their high potential for orgasm can be used to improve their sexual satisfaction.

This study showed that a significant portion of transgender women were not familiar with any STIs signs and symptoms and unfortunately 88.2% did not even consider themselves at risk for HIV infection. This finding is mostly related to cultural factors and lack of awareness on STIs in traditional societies such as Iran. In India for instance, 88% of transgender people were not familiar with STIs (8) but in Canada 95% of transgender people were familiar with at least three main ways of HIV transmission [34].

STIs and HIV screening was never performed in 87.4% and 72.4% of participants respectively. However, among the few transgender women who underwent these tests, 1.6% were HIV positive and 18% had STIs. However, a study on general population of Iranian women indicated that the prevalence of gonorrhea is 0–2.4%, chlamydia 6.4–10.3%, syphilis less than 1%, HPV 7% and the prevalence of HIV infection is 0.14% [35]. The high prevalence of STIs and HIV in transgender women compared to the general population indicates their high vulnerability to high-risk sexual behaviors such as unprotected sex and sometimes having multiple sex partners. Similarly, in the studies conducted on transgender women, the prevalence of STIs was between 13 and 21% [22, 24, 36]. In contrast to the present study, in most studies, the average prevalence of HIV infection in transgender women based on a positive laboratory test was between 14 and 41% [21, 22, 36, 37], which is significant, because more than 72% of participants in this study had never undergone HIV test. Also, these people have been deprived of other health services due to social stigma and discrimination [11].

In this study, about one-third of participants never used condoms during sex, and half of them used it occasionally. In addition, they mentioned that the most common reason for not using a condom was not having one, and given that the majority of participants in the study were in poor financial conditions, it seems necessary to provide them with free condoms and give them necessary training for safe sex. Results of other studies showed that the average condom nonuse in transgender people was low and between 34 and 48% [22, 36, 38].

To interpret the results of this study, it is necessary to consider its limitations such as data being self-reported and the lack of a cisgender group for comparison. Also, information on sexual behavior variables such as sexual satisfaction, body satisfaction, etc. has been collected by a single question. However, this is the first study in Iran that examines sexual behaviors and vulnerability to sexually transmitted diseases in transgender women in a significant sample size.

## Conclusion

Results of this study indicated that the majority of transgender women had sex experience in their life despite being single. Some of them were involved in high-risk sexual behaviors such as not using condoms and multiple sexual partners. In addition, lack of awareness about the symptoms and complications of STIs and ways of transmitting HIV exposes these people to STIs. In order to improve the sexual health of transgender women, special attention should be paid to sexual counseling, especially with their sex partner, as well as raising their awareness on STIs can be very effective in reducing high-risk behaviors.

In terms of sexual feelings, transgender women, despite having orgasm, had little satisfaction with both their appearance and their sex life, and believed that body satisfaction greatly affected their sexual satisfaction. Also in this study, a significant relationship was observed between vaginoplasty and sexual satisfaction. Given that a high percentage of the subjects in the present study had not yet undergone vasinoplasty, the need for gender affirming surgeries and psychiatric interventions affecting body satisfaction was identified in this group.

## Data Availability

The data are available from the corresponding author on reasonable request.

## Abbreviations

STIs: Sexually transmitted infections
CVI: Content validity index
CVR: Content validity ratio
